# Investigation of the Effect of Temperature on the Wear Rate and Airborne Noise in Sliding Wear

**DOI:** 10.3390/ma15030812

**Published:** 2022-01-21

**Authors:** Kevin Lontin, Muhammad Khan, Bander Alharbi

**Affiliations:** School of Aerospace, Transport and Manufacturing, Cranfield University, Cranfield MK43 0AL, UK; Muhammad.A.Khan@cranfield.ac.uk (M.K.); Eng.Banderh@hotmail.com (B.A.)

**Keywords:** friction, wear, airborne noise

## Abstract

When friction processes occur, wear is generated. The generation of wear also leads to airborne noise. There have been many research studies on wear and its correlation with airborne noise, but most research has focused on experimental aspects, and theoretical models are rare. Furthermore, analytical models do not fully account for the wear and airborne noise generation, especially at an asperitical level. One model was developed that gave a reasonable quantification for the relationship between wear and airborne noise generation at an asperitical level under room temperature. In this paper, the accuracy of the model is assessed at higher temperatures. Two materials were set up on a tribometer (aluminium and iron) at 300 RPM. The samples were tested at two different temperatures (40 and 60 degrees) and two different loads were applied (10 N and 20 N). The model computed the predicted wear and sound pressure, and it was compared with the experimental results. The errors are larger for the wear than when the model was validated at room temperature. However, the increase in the error for the sound pressure was smaller at higher temperatures (approximately 20–30%). This is due to the assumptions that were made in the initial model, which are exacerbated when higher temperatures are applied. For example, flash temperatures were neglected in the original model. However, when initial heat is applied, the effects of flash temperatures could be more significant than when no heat is applied. Further refinements could improve the accuracy of the model to increase its validity in a wider temperature range.

## 1. Introduction

Wear in machine components is vital in the industry as it leads to material failure and a loss of the component’s functionality, which cannot be detected easily especially in complex systems. Moreover, detecting and replacing these damaged components is expensive and time consuming. Friction and contact surface wear are caused by the relative motion between two bodies, which can have a detrimental impact on mechanical system dependability, security, and use [1]. The volume loss from solid surfaces in moving contact is what is measured as wear [2,3]. To increase the life of a mechanical system, it is necessary to distinguish and anticipate wear states. It is, however, hard to measure the wear states of complex nonlinear dynamic systems [1].

The severity of surface deterioration can significantly alter the noise spectrum, therefore analysing the noise emitted may lead to a probable way for diagnosing or detecting fault components in mechanical systems [4]. Roughness noise is produced when two surfaces slide against each other [5]. The magnitude and frequency of the noise are directly related to the surface wear of the contact area [6]. The correlation between the wear and noise generated is not a new phenomenon in machine health diagnosis and has been established since the 1970s where the noise signals were used and the effectiveness of noise signals was evaluated mostly on rotating machines and industrial tool wear, with their trend spectra being analysed to discover the relationship between noise and wear [4,6]. Acoustic emission is a stress wave that passes through a material as a result of a rapid release of strain energy. In recent years, acoustic emission (AE) tools and systems have been developed for non-destructive testing and monitoring of the structural integrity and general quality of a range of materials, manufacturing processes, and other crucial devices [7].

Numerous experimental studies support the use of AE-based methodologies and other non-destructive techniques to diagnose or detect fault components in mechanical systems, particularly wear monitoring [8,9,10,11,12,13]. Chen et al. [14,15] investigated the link between the tribo-surface and friction coefficient, as well as the sliding speed and friction noise, using a reciprocating system. Similarly, Jibiki et al. [16] employed a reciprocating system to investigate the relationship between the amount of wear and the friction coefficient on tribo-surface noise. Kong et al. [17] used support vector regression to build a predicted model for the wear and then compared the emitted signal to the real wear. Serrao et al. [18] employed the Taguchi approach to predict wear behaviour when the load, speed, sliding duration, and signal-to-noise ratio were all varied. Under varying operating settings, the observed results revealed a strong link between wear and acoustic emissions. Feng et al. [19] presented a structured review of past studies on the effects of tribological and contact-dynamic parameters on AE signal features, as well as a summary of all the key empirical findings in terms of correlations between contact dynamic parameters, wear, and AE signal parameters. The wear of components is also affected by load and temperature. As the load and temperature increase, the wear rate increases until a certain temperature is reached [20,21]. For example, Pei et al. investigated the effect of temperature for GCR15 sliding against NM600 from room temperature to 300 °C. The wear rate increased rapidly then decreased as the temperature continued increasing [22].

The tribological behaviour of composite materials also follows the same trend. The wear resistance of composite materials was found to decrease as the temperature increased [23]. This is due to the fact that, on metallic and composite surfaces, when temperatures reach a certain value, a protective layer is formed between the contacting surfaces. Therefore, this leads to a reduction in wear rate [24]. Other possible causes for the reduction of wear rate at very high temperatures include the change in wear mechanism [25]. The effect of temperature on fretting wear was also examined. The results correlated with results from previous research. In fretting wear, there is a transition temperature, which depends on the fretting frequency. Before the transition temperature, the wear rate increases rapidly. However, after the transition temperature, the wear rate starts to decrease [26]. Pearson et al. investigated the effect of the formation of the oxide particles due to fretting wear at high temperatures. It was found that those oxide particles would contribute to the lower wear rate at very high temperatures. This was investigated over a wide range of temperatures from 24 °C to 85 °C [21]. Other experiments performed on nickel-based alloys also show that the formation of a layer of oxide during high-temperature fretting wear acts as a protective third body between the contacting surfaces [27]. Nassar et al. investigated the effect of cold temperatures on metallic alloys reinforced with ceramic particles. As expected, colder temperatures decrease the wear rate of the materials [28]. Furthermore, the effect of lubrication at high temperatures was also investigated. This is because such investigations are very important, especially in the automotive industry where high temperatures, such as on brake pads, are very common [29]. There have also been numerical models used to assess the effect of temperature on the wear rate. One such example is the modelling of the wear resistance of a 2014 Al alloy reinforced with Al_2_O_3_ ceramic particles. Both the finite element analysis and the experimental results showed an increase in wear rate for temperatures up to and including 170 °C [30].

The effect of temperature on friction-induced noise has also been thoroughly investigated. Matozo et al. studied the effect of both temperature and humidity on brake pads squeal noise and it was found that altering the humidity and the temperature would also change the sound spectrum [31]. A similar study was performed by Mahale et al. for temperatures ranging from 50° C to 200 °C where they correlated the change in the squeal noise of the brake systems to the change of the thermoelastic behaviour of the brake material [32]. Other research found that, specifically for the automotive industry, the relationship between the friction processes between the tires and the pavement and the sound generation followed a linear pattern [33].

Recently, the Khan–Lontin model was developed (i.e., a fully analytical model) that attempts to quantify the interdependencies between the wear and the airborne noise emitted during a sliding friction process with no lubrication [34]. The model was validated at room temperatures. However, it was not validated for higher temperatures. At present, there are no general analytical models that incorporate the wear and noise at high temperatures. In this paper, the accuracy of the model is assessed when a high initial heat input is applied to the samples. This is important because many wear processes will increase the temperatures of the system, and validating the model for higher temperatures would increase the applicability of the model.

## 2. Theory

**Figure 1:**
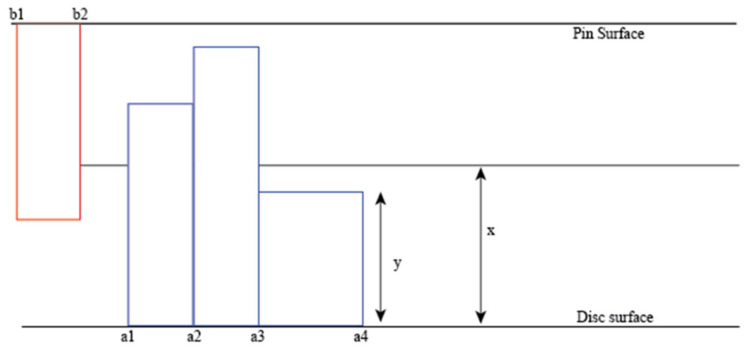
Conditions of contacts. a_n_ and b_n_ are points on the disc surface and pin surface, respectively. y is the height of asperity (µm) and x is the distance between the surface and the centreline (µm), which is defined as the mid-point between the two surfaces. Reproduced from Ref. [34].

The analytical model used to quantify the interdependence between the wear and the airborne noise emitted during a sliding friction process was created based on the conditions of contacts shown in Figure 1.

In general, the asperities will be found in one of three regimes as sliding wear occurs. This depends on the contact forces. If the contact forces cause the asperities to remain in the elastic zone, then the asperities would vibrate elastically. If the contact forces exceed the yield strength of the asperities, then the asperities would be plastically deformed and thus would not vibrate. Finally, if the contact forces exceed the ultimate tensile strength of the asperities, then they would break and that would lead to wear. The same assumptions for room temperatures were assumed to hold true. Those assumptions are as follows:

1. The asperities are considered to behave as macroscopic cantilever beams.
2. Asperities act independently of one another. This means that each asperity struck does not influence what happens to the next asperity.
3. Macroscopic material properties are observed. This is because the asperities are on a mesoscopic scale and so macroscopic behaviour is a reasonable approximation to make.
4. The asperities do not undergo elastoplastic deformation. This is because elastoplastic behaviour does not contribute significantly to the wear or the noise.
5. Only one asperity was modelled on the pin.
6. The disc is not modelled as a continuous counter-profile.

Additionally, the wear function remained the same. The wear function is the function *W*(*y* − *x*), such that the following equation holds:

(1) $$\phi \left(y-\Delta y,\;t+\Delta t\right)=\Phi \left(y,t\right)*W\left(y-x\right),$$

A partial differential equation can be formed as shown in the following equation:

(2) $$\frac{\partial }{\partial t}\Phi \left(y,t\right)=-\frac{\partial }{\partial y}\left(W\left(y-x\right)\Phi \left(y,t\right)\right)$$

The solution to the differential equation is:

(3) $$\Phi \left(y,t\right)=\frac{W\left[\theta \left\{\varphi \left(z\right)-t\right\}\right]}{W\left(z\right)}exp\left(G\left[x+\theta \left(\varphi \left(z\right)-t\right)\right]\right)$$

where *Δy* is the height changes of the asperities, *Φ*(*y* − *Δy*, *t* + *Δt*) is the asperity height distribution at *t* + *Δt,* and *Φ*(*y*,*t*) is the asperitical height distribution at time *t*.

(4) $$\varphi =\int \frac{1}{W\left(z\right)}dz$$

*θ* is the inverse function such that *θ*(*φ*(*z*)) = *z* and *G* = log(*Φ*(*y*,0)).

**Figure 2:**
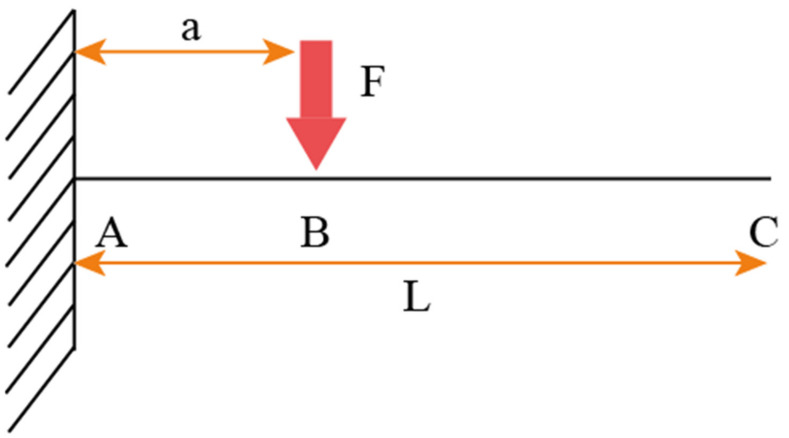
Forces acting on a cantilever beam. Reproduced from Ref. [34].

The wear function was calculated using the beam theory as shown in Figure 2.

It is given by the following equation:

(5) Wz=Cz12

where *A* is the point of the asperity located on the substrate, *C* is the endpoint of the asperity and *B* is the point at which the impact occurs. a and L are the distance from *A* to *B* and the total height of the asperity respectively. In Equation (5), *C* = *A* + *B* and

(6) A= 3EIθS12

(7) B= 2EIθS3EIθS12

where *E* is the elastic modulus, *θ* is the stress, *s* is the section modulus, and *I* is the second moment of area. Using the wear function in the analytical model allows the prediction of wear and noise given a set of initial operating conditions. The model was originally only validated at room temperatures. To assess the accuracy of the model at high temperatures, a set of validation experiments was performed using a pin-on-disc setup under varying loads at 300 RPM.

## 3. Methodology

In order to assess the accuracy of the experiments, an experimental scheme was devised to perform a set of validation experiments under varying operating conditions at varying loads and temperatures. The experiment scheme is shown in Table 1.

**Figure 3:**
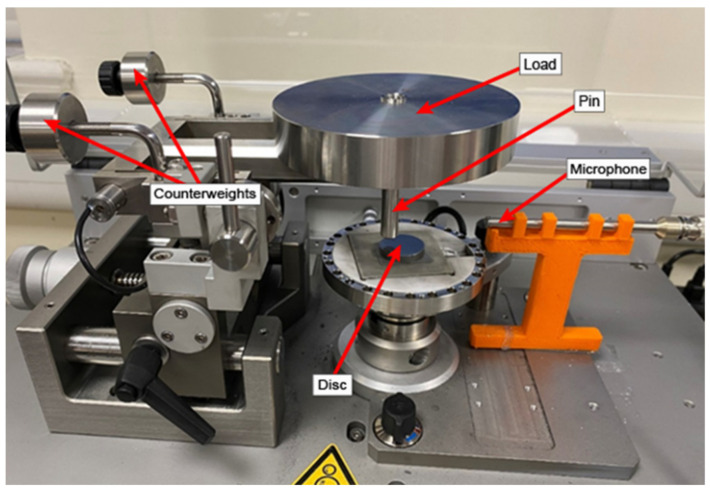
Close-up of the tribometer. Reproduced from Ref. [34].

The experimental scheme was chosen in order to assess the validity of the model under varying operating conditions. There were three main important factors that could affect the results. The first factor was the choice of materials. This is very important as different materials have different wear rates. Iron (hardness of 150 HV) and aluminium (hardness of 15 HV) are common materials used in a wide range of industries, so they were a good choice for the validation experiments. The sample discs were prepared and polished in order to give them a relatively consistent surface roughness. In order to do so, they were placed in resin and a machine was used to grind them with 800 grit paper at a constant pressure. The samples were then carefully removed from the resin to prevent damage to the discs. Two different loads were chosen to determine the impact of load on the wear rate and the sound generation during the friction processes. Finally, the temperature was altered to investigate the effect of temperature on the wear rate and sound generation. All those parameters were altered in each of those 12 sets of experiments. The sliding speed remained constant at 300 RPM, however. The tribometer is shown in Figure 3.

**Figure 4:**
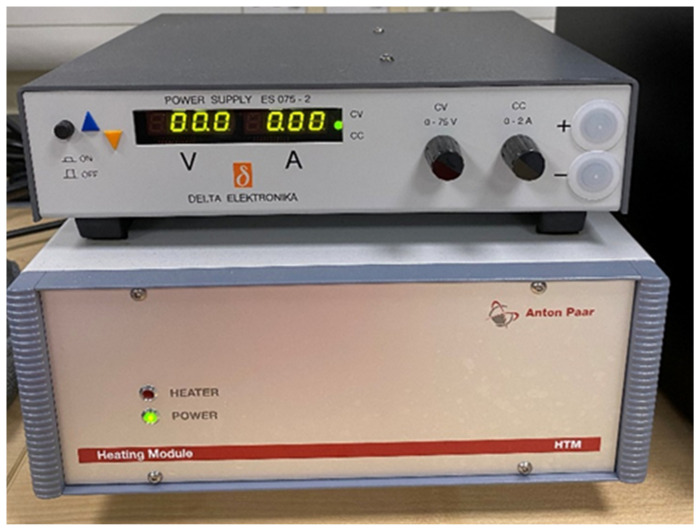
Tribometer heating unit.

The tribometer used was an Anton Paar TRB3 tribometer (Anton Paar, Graz, Austria) with an integrated wear depth sensor. The acquisition rate on the wear depth sensor was 100 Hz. The counterweights are used to balance the weight of the pin such that the pin will not act as an extra load. The load is placed on top of the pin and acts as the normal force on the samples. Two loads were used for the validation experiments: 10 N and 20 N. A stationary 3-mm-radius pin (stainless-steel 440C with a hardness of 555 HV) was set up on a rotating disc. The pin was assumed to not wear out, and in order for this to be consistent, it was checked for signs of wear and replaced between each experiment. A rubber O-ring was added to minimise external sources of vibrations. A GRAS pressure microphone (GRAS Sound & Vibration, Copenhagen, Denmark) was used with a maximum operating frequency of 20 KhZ. The dynamic range of the microphone ranged from 17 dB to 138 dB and the sensitivity was set at 47.46 mV/Pa. The microphone was connected to a DAQ card (NI9174) (National Instruments Corporation, Austin, TX, USA), which was connected to a computer in order to record the sound data. The penetration depth of the tribometer was used to calculate the wear. In order to apply the initial heat input to the samples, a heating unit was connected to the tribometer. The error between the indicated temperature on the heating unit and the real temperature as measured on the disc sample was 0.3 °C. This is shown in Figure 4.

**Figure 5:**
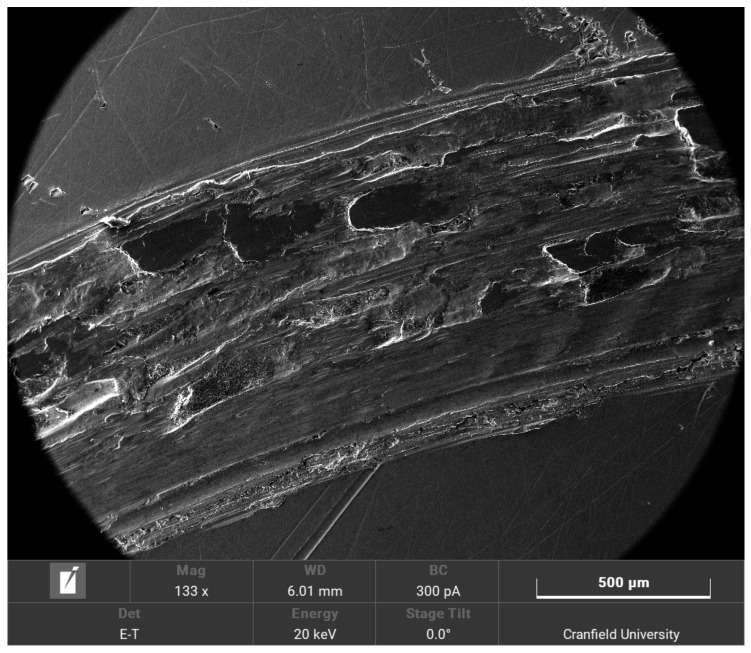
SEM image of the iron sample.

**Figure 6:**
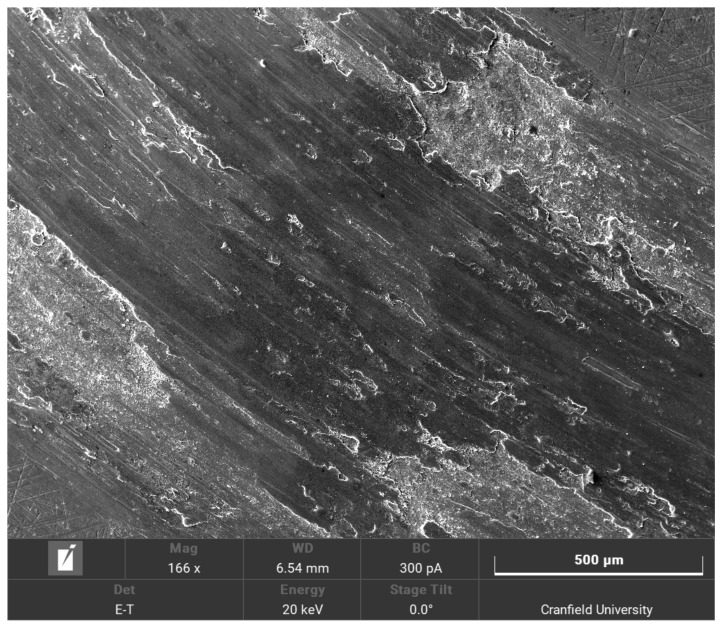
SEM image of the aluminium sample.

Each experiment cycle lasted for 10 min at 300 RPM. Each material was tested three times at both 40 °C and 60 °C under both 10 N and 20 N loads and the average results were taken. Prior to the experiments, the consistency of the surface roughness of the samples was taken. Interferometry measurements were performed on randomly selected samples. They were performed at 3 random points on each sample and the average surface roughness was calculated. It was found to be at around 0.5 µm. At the end of the experiments, a set of SEM images were taken of randomly selected samples in order to visualise the wear. One of the iron samples at 60 °C is shown in Figure 5 and one of the aluminium samples is shown in Figure 6.

The main mechanism of wear that occurred during the experimental validation is abrasive wear. Adhesive wear would occur at higher temperatures. However, the temperatures used in the experimental validation were kept low enough so that the wear mechanism would not change. This is because the model is only valid for abrasive wear where the material is removed. Adhesive wear mechanisms (where the material is transferred between the two surfaces) would invalidate the model.

## 4. Results

**Figure 7:**
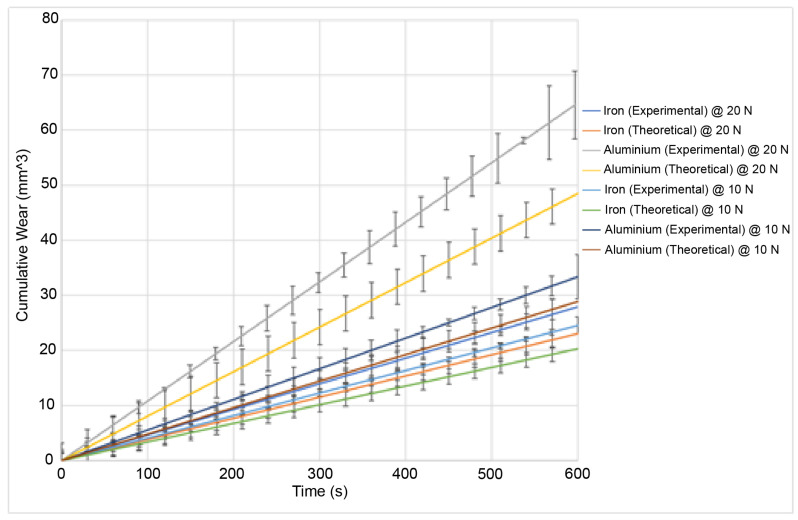
Experimental results for the wear at a 20 N load and 40 °C for aluminium (grey) and iron (blue). Theoretical results at a 20 N load and 40 °C for aluminium (yellow) and iron (orange). Experimental results at a 10 N load and 40 °C for aluminium (violet) and iron (teal). Theoretical results at a 10 N load and 40 °C for aluminium (brown) and iron (green).

**Figure 8:**
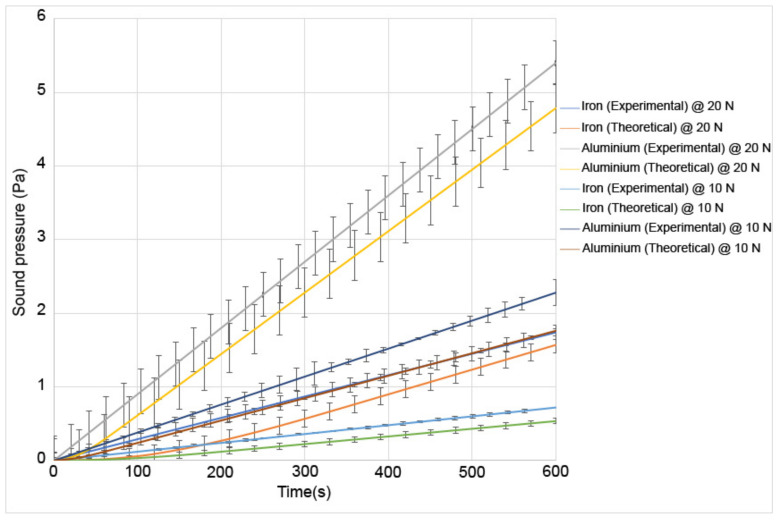
Experimental results for the sound pressure at a 20 N load and 40 °C for aluminium (grey) and iron (blue). Theoretical results at a 20 N load and 40 °C for aluminium (yellow) and iron (orange). Experimental results at a 10 N load and 40 °C for aluminium (violet) and iron (teal). Theoretical results at a 10 N load and 40 °C for aluminium (brown) and iron (green).

The wear and sound pressure at an initial temperature of 40 °C are shown in Figure 7 and Figure 8.

**Figure 9:**
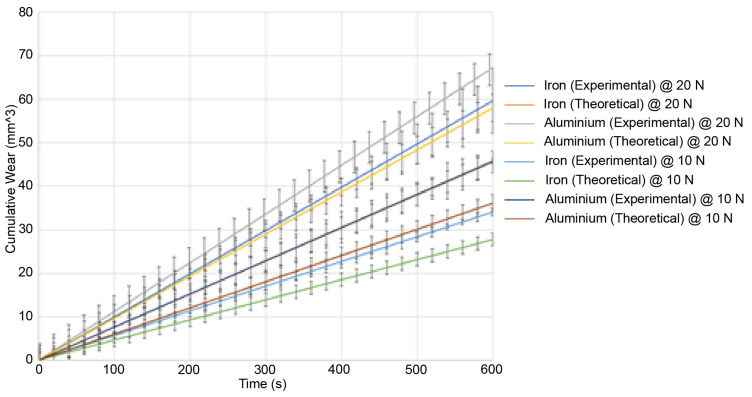
Experimental results for the wear at a 20 N load and 60 °C for aluminium (grey) and iron (blue). Theoretical results at a 20 N load and 60 °C for aluminium (yellow) and iron (orange). Experimental results at a 10 N load and 60 °C for aluminium (violet) and iron (teal). Theoretical results at a 10 N load and 60 °C for aluminium (brown) and iron (green).

It can be seen from the two figures that a higher load leads to higher wear as shown by the results from the experiments under a 20 N load. This also correlates to a higher sound pressure as shown in Figure 9. From the experimental results, aluminium undergoes the highest wear at 40 °C followed by iron. Aluminium also has the highest sound pressure, with iron having the lowest sound pressure. The results under 10 N show that a lower load leads to both lower wear and a lower sound pressure. The results at 60 °C show that a higher temperature leads to higher wear whereas a lower temperature will lead to lower wear. The trends are similar in that a higher load will also lead to both a higher wear and sound pressure regardless of the initial temperature. At high temperatures, aluminium undergoes the highest wear and iron undergoes the lowest wear. The analytical results reflect that trend too [34]. However, the errors are larger at higher temperatures and higher loads.

**Figure 10:**
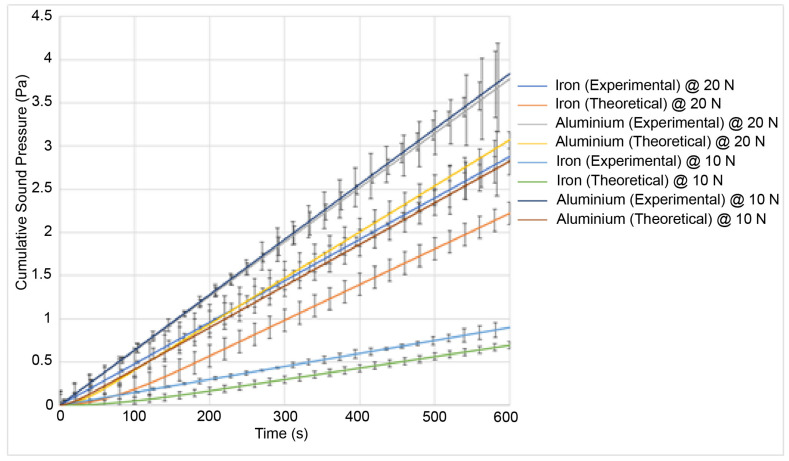
Experimental results for the sound pressure at a 20 N load and 60 °C for aluminium (grey) and iron (blue). Theoretical results at a 20 N load and 60 °C for aluminium (yellow) and iron (orange). Experimental results at a 10 N load and 60 °C for aluminium (violet) and iron (teal). Theoretical results at a 10 N load and 60 °C for aluminium (brown) and iron (green).

The wear and the sound pressure at an initial temperature of 60 °C are shown in Figure 9 and Figure 10:

## 5. Discussion

### 5.1. Predicted and Observed Wear

The error is larger at high temperatures than they are at lab temperatures [34], between 20–30%. This is due to the limitations of the model. The model was simplified in order to reduce the computational costs, and several assumptions were made. One of the major assumptions that were made was the modelling of the top asperity (the asperity that does not wear out). The asperity causes a point impact on the bottom asperities. This is a simplification, as in reality, it would be a distributed impact force across the length of the contacting asperities. Furthermore, since a point impact was assumed, it might have been a better idea to model the non-wearing asperity as a mass-spring system. This is reinforced by the fact that the top surface is modelled as a single asperity only, which contacts one asperity on the bottom surface at any moment in time. However, in reality, there would be multiple contacts between the asperities on the pin surface and the asperities on the disc surface. However, modelling a full set of asperities on both surfaces and increasing the number of contact points would increase the complexity of the model exponentially. Moreover, due to uncertainties in the model, the asperities are regarded as independent of one another. As the temperatures are increased, the vibrational velocities of the asperities are also higher as they have a higher energy input. If the vibrational velocities are higher, the likelihood of those asperities interacting with one another increases and this reduces the accuracy of the model. This effect was neglected because incorporating this introduces too many uncertainties in the model as the exact interactions between vibrating asperities cannot be readily ascertained. Furthermore, due to higher initial temperatures, flash temperatures become more important. Flash temperatures are short-lived sudden increases in temperatures that occur due to the sliding friction processes. Those temperatures occur when asperities are struck since, according to the theoretical model, not all of the energy is released as sound. Roughly half of the energy is released as heat. Due to the heat that is already initially applied to the sample, the combined effect of the applied heat and the flash temperatures do increase the wear rate. The model does not account for those. As the wear is already underestimated even with the absence of heat, the application of heat increases the error. Finally, the asperities were also assumed to behave as macroscopic cantilever beams. This is due to the fact that the model uses the beam theory to calculate the bending and vibrations of the asperities. Macroscopic material properties used as the scale of the asperities were deemed sufficient so that macroscopic material properties would hold. The general trend of the results, however, are in agreement with existing research. As the temperature increases, the wear rate also increases [22]. The main mechanism observed in the experimental validation is abrasive wear. This is the same mechanism as the experiments performed under room temperature conditions. This is because, although the temperatures are higher, they are not high enough to cause a change in the wear mechanism. At even higher temperatures, adhesive wear and oxidation wear would become the dominant mechanisms. Oxidation wear was not observed in this case. This explains the increase in the wear rate. As the temperature continues increasing and oxidation wear becomes a main component of the wear mechanism, a reduction in the wear rate would be observed. This is due to the fact that the oxide layer is usually a harder surface than the disc substrate. It would thus act as a protective third body, thus reducing the impact of the sliding wear on the underlying surface. The model does not account for third-body layers as it assumes direct asperitical contacts. As such, the formation of an oxide layer would reduce the accuracy of the model even further. The model would cease to be valid at such high temperatures.

### 5.2. Predicted and Observed Sound Pressure

The sound pressure generated is higher under a higher load than under a lower load. This is consistent with the existing research as a higher sound generation is correlated to higher wear. However, it should be noted that adhesive wear and abrasive wear both have different mechanisms for sound generation [35]. The model is only valid where the dominant mechanism is abrasive wear. Adhesive wear features high-frequency peaks whereas abrasive wear features lower-frequency peaks. At higher temperatures (reaching around 100 °C and above), the change in the wear mechanism would lead to a change in the sound spectrum such as a shift in frequencies. The model does not account for those, and this is why it is constrained in temperatures below 100 °C. It is reasonable to assume that as the sound increases, so does the wear. The error for the sound pressure between the theoretical and the experimental results is around 20%. This is lower than for the wear. This is due to the ductility of the materials at high temperatures. There are two contributors to the sound pressure: The elastic vibrations and the wear. The wear is a larger contributor than the elastic vibrations of the asperities. This is why as the wear increases, the sound pressure also increases. However, the size of the plastic zone affects the sound generation. If the plastic zone is large and the asperities enter the plastic zone quicker at high temperatures, then fewer asperities are elastically vibrating at any given time and they remain in the plastic zone for long enough that, despite the increase in wear, enough asperities are in the plastic zone at any given time and this introduces an offset in the sound pressure generation as asperities in the plastic zone do not contribute to the sound pressure. This means that the higher wear has less of an impact on the sound generation at a higher temperature than at a lower temperature. This explains the lower error in the theoretical model. Out of the two materials tested (iron and aluminium), iron generates the least sound and aluminium generates more sound. This is also consistent as aluminium undergoes the highest wear and iron undergoes the least wear. This is consistent with existing research as iron is more wear resistant than aluminium. The sound increase is also mostly linear. Finally, it should be noted that the sound shown in the results is the cumulative sound pressure, similar to the results for the wear, which show the cumulative wear. This was performed for ease of comparison.

## 6. Conclusions

This paper presents the validation of the analytical model at high temperatures. Previously, the analytical model was validated at room temperatures, but the effects of high temperatures were neglected. In this paper, the accuracy of the analytical model was assessed at higher temperatures. Predictably, it is found that the wear is higher at higher temperatures and under a higher load. Higher wear also correlates to a higher sound pressure. This is consistent with existing research. However, the accuracy of the analytical model is reduced at higher temperatures as the errors for both the wear and the sound are greater. The analytical model has many assumptions that were made in order to reduce the complexity of the model at the expense of accuracy. At high temperatures, this is exacerbated. For example, the effects of flash temperatures are more significant but were neglected in the model. Further refinements to the analytical model could account for some of these inaccuracies such as modelling the top asperity as a mass-spring system, which could potentially be an improvement to the current cantilever beam model. Finally, a scaling factor could be applied to the model in order to account for the mostly linear error between the experimental results and the analytical results. This would mean that the model would be a combination of empirical modelling and analytical modelling for the final results, but it would be a more immediate way to solve these inconsistencies.

## Figures and Tables

**Table 1:** Experimental scheme.

Set No.	No. of Samples	Material	Load (N)	Temperature (°C)
1	3	Iron	10	40
2	3	-	-	60
3	2	-	20	40
4	3	-	-	60
5	3	Aluminum	10	40
6	3	-		60
7	3	-	20	40
8	3	-	-	60

## Data Availability

The data presented in this study are available on request from the corresponding author.
